# Species delimitation and digit number in a North African skink

**DOI:** 10.1002/ece3.326

**Published:** 2012-10-24

**Authors:** R P Brown, T Tejangkura, E H El Mouden, M A Ait Baamrane, M Znari

**Affiliations:** 1 School of Natural Sciences and Psychology, Liverpool John Moores UniversityLiverpool, L3 3AF; 2 Department of Biology, Faculty of Science – Semlalia, Cadi Ayyad UniversityMarrakech, 40000, Morocco

**Keywords:** Methods, molecular ecology, population genetics

## Abstract

Delimitation of species is an important and controversial area within evolutionary biology. Many species boundaries have been defined using morphological data. New genetic approaches now offer more objective evaluation and assessment of the reliability of morphological variation as an indicator that speciation has occurred. We examined geographic variation in morphology of the continuously distributed skink *Chalcides mionecton* from Morocco and used Bayesian analyses of nuclear and mitochondrial DNA (mtDNA) loci to examine: (i) their concordance with morphological patterns, (ii) support for species delimitation, (iii) timing of speciation, and (iv) levels of gene flow between species. Four digit individuals were found at sites between Cap Rhir (in the south) and the northern extreme of the range, whereas five-digit individuals were found in two disjunct areas: (i) south of Cap Rhir and (ii) the north of the range where they were often syntopic with four-digit individuals. The pattern of variation in generalized body dimensions was largely concordant with that in digit number, suggesting two general morphotypes. Bayesian analyses of population structure showed that individuals from sites south of Cap Rhir formed one genetic cluster, but that northern four- and five-digit individuals clustered together. Statistical support for delimitation of these genetic clusters into two species was provided by a recent Bayesian method. Phylogenetic–coalescent dating with external time calibrations indicates that speciation was relatively recent, with a 95% posterior interval of 0.46–2.66 mya. This postdates equivalent phylogenetic dating estimates of sequence divergence by approximately 1 Ma. Statistical analyses of a small number of independent loci provide important insights into the history of the speciation process in *C. mionecton* and support delimitation of populations into two species with distributions that are spatially discordant with patterns of morphological variation.

## Introduction

Traditionally, most species have been described using morphology, but morphological analyses cannot easily demonstrate isolation and so genetic analyses are generally required (Wiens 2007). Statistical techniques that estimate key parameters associated with population sizes, divergence times, and assess concordance between gene trees have further encouraged the use of molecular sequence data for establishing species boundaries (e.g., Nielsen and Wakeley 2001; Yang and Rannala 2010). Specifically, the program BPP (“Bayesian Phylogenetics & Phylogeography”: Yang and Rannala 2010) provides a test of whether species can be delimited genetically using a Bayesian coalescent method that examines splits in multiple gene trees relative to a user-defined guide tree. It also accommodates uncertainty in the gene trees (as well as stochastic fluctuations in the coalescent process) which may be useful for intraspecific analyses of loci that contain relatively little genealogical information. Analyses of sequence data simulated under a two species model show that this method has great power to correctly delimit the species with only 1–2 loci when >5 individuals are sampled from each (Zhang et al. 2011). One criticism of statistical genetic species delimitations is that they lack weight without additional support from independent data sets describing other important characteristics such as morphology or ecology (Bauer et al. 2011). Comparisons between genetic delimitations with morphological variation and ecology/geography should help reveal the consistency between these alternative sources of information.

Additional approaches can provide other important insights into speciation. Algorithms implemented within programs such as IMa2 (“Isolation with Migration 2”:Hey and Nielsen 2007; Hey 2010) jointly examine migration and divergence times of two or more genetic lineages. This approach may be useful because metapopulation lineages that separated a long time ago and subsequently exchanged relatively few genes are more likely to merit full species status. Rigorous estimates of the timing of the population split also require a Bayesian approach. The simple model in IMa2 may provide suitably accurate estimates, but many real data sets lack sufficient information to allow estimation of both migration and timing of the ancestral population split (Hey 2010). Also, the model does not incorporate rate variation between lineages (Hey and Nielsen 2004). Hence, when conditions allow, it might be preferable to use a phylogenetic approach that incorporates time calibration information on a tree containing the populations of interest (Thorne and Kishino 2002; Yang and Rannala 2006). Phylogenetic dating does not incorporate migration and so might be more suitable when migration is low. However, until recently, this approach dates sequence divergence times rather than species divergence times. The latter may postdate the former by quite a significant amount when speciation occurred recently. A new approach that overcomes these difficulties by taking account of the ancestral coalescent process has been implemented within the program *BEAST (“Bayesian Evolutionary Analysis by Sampling Trees”:Drummond and Rambaut 2007; McCormack et al. 2010). This combined phylogenetic–coalescent approach may represent a significant advance in estimation of species divergence times on shallow phylogenies.

Here, we examine populations of the skink *Chalcides mionecton* Böttger 1874 which is found along ∼900 km of the Atlantic coast of Morocco. It is mainly restricted to coastal habitats but has been cited up to 150-km inland (Bons and Geniez 1996). Geographic variation in digit number has been used to describe two subspecies. *Chalcides mionecton mionecton* Böttger 1874 will be referred to as the northern form because it is applied to populations from Tangiers in the north to Cap Rhir ∼700 km to the southwest (Schleich et al. 1996). It is characterized by possession of four digits on both the manus and the pes. The southern form, *C. mionecton trifasciatus* Chabanaud 1917, has five digits on the manus and the pes and has been recorded from Cap Rhir south to Foum Assaka (Bons 1959). Known deviations from this categorical north–south pattern include a northern locality, Mehdia Plage, where five digit individuals are found (Mateo et al. 1995). Southern individuals with four digits have also been documented (Pasteur 1962). Northern and southern forms are not separated by any obvious physical barriers, although Schleich et al. (1996) suggested that the narrowing of the coastal plain at Cap Rhir reduced gene flow between them.

Gain and loss of digits occurs frequently among scincid lizard lineages (e.g., Pasteur 1981; Greer 1987; Kohlsdorf and Wagner 2006; Brandley et al. 2008; Skinner et al. 2008; Siler and Brown 2011). Anatomically, four-toed *C. mionecton* possess fifth metatarsal/metacarpal bones but lack distal or proximal phalanges for this digit (Raynaud et al. 1989). In squamates, changes in numbers of digits can be associated with body elongation and limb reduction (Wiens and Slingluff 2001). This trend toward a snake-like body in some lizards seems to be associated with the evolution of either a burrowing or surface-dwelling ecomorph (Wiens et al. 2006).

The pattern of digit variation in *C. mionecton* could reflect independent evolution in lineages that diverged some time ago. In this case, concordant splits are expected at multiple independent loci. Alternatively it could reflect natural selection without lineage divergence and therefore solely affect loci associated with digit number. A comprehensive phylogenetic analysis of European and African *Chalcides* included both subspecies of *C. mionecton* (Carranza et al. 2008). It demonstrated reciprocal monophyly between them, supporting lineage divergence. However, this was a cross-species mitochondrial DNA (mtDNA) study and therefore only analyzed some of the genetic diversity within *C. mionecton,* making this conclusion tentative.

*Chalcides mionecton* represents an interesting model for investigating speciation in relation to morphological evolution. Here, we examine (i) the evidence for genetic species within *C. mionecton*, (ii) whether these reflect morphological differences, and (iii) the timing of divergence and degree of gene flow between these putative species.

## Materials and Methods

### Specimens and sites

**Figure 1:**
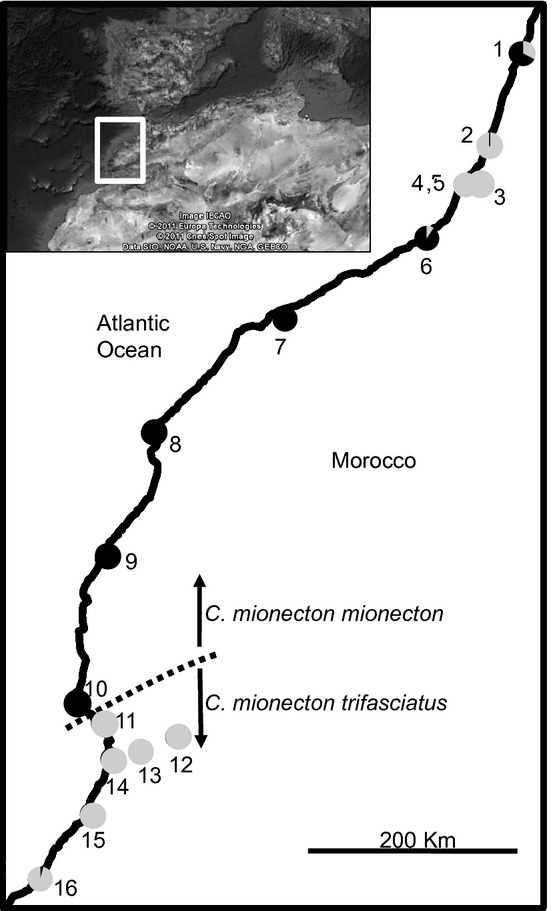
Sample sites, pie charts with proportions of four digits (black) and five digits (gray) at sites, and subspecies of *Chalcides mionecton*. (Note that both subspecies were described from Cap Rhir, site 10, by previous work (Schleich et al. 1996), but only the northern subspecies was found by this study).

A total of 192 *Chalcides mionecton* were captured from 16 sites extending from the north to the south of its range during field trips carried out between June 2006 and June 2008 (Fig. 1, Appendix S1). Sites were quite evenly spaced, apart from northern sites 2–5. (This was because all individuals from site 5 had five digits and so 1–3 specimens were obtained from neighboring areas to explore the extent of this form.) DNA was extracted from tail tips that were removed from 102 specimens, stored in ethanol, and then one or more loci were sequenced from these individuals (see later). Captured specimens were euthanized by injection of pentobarbital, fixed in formaldehyde, and deposited in the Natural History Museum of Marrakech.

Canary Island skinks belong to the same Western *Chalcides* clade as *C. mionecton* (Carranza et al. 2008). Divergence times between the Canary Islands of La Gomera and El Hierro are known, providing a time calibration for divergence within *C. mionecton* (see below). We used individuals that had been collected for previous research projects (e.g., Pestano and Brown 1999; Brown et al. 2000); see Brown and Pestano (1998) for more information. One individual was used from each site, unless otherwise stated: *Chalcides viridanus* from Guaza and Igueste in Tenerife, *C. sexlineatus tus* from Tafira Baja (*n* = 2) and Juan Grande in Gran Canaria, and *C. coeruleopunctatus* from Calera and Vallehermoso in La Gomera and from Valverde and Frontera in El Hierro.

### Morphology

External morphology of the 192 museum specimens was recorded. The numbers of digits was recorded from both the manus and the pes. The following 12 body dimensions were measured to the nearest 0.1 mm using digital calipers: snout–vent length (SVL), head length (HL), head depth (HD), head width (HW), jaw length (JL), snout–thorax (ST), snout–hind limb length (SHL), fore–limb length (FLL), rear–limb length (RLL), fourth toe on fore–limb length (FTFL), fourth toe on hind limb length (FTHL), and body width at thorax (BWT). The following three scalation characters were also recorded: the number of ventral scales rows between the rostral scale and the cloaca, the number of scales counted around the mid-trunk, and the number of infra-digital lamellae on the fourth toe of the hind limb. (Postnasal, supralabial, supraciliary, supranasal, and frenocular scales were recorded but not analyzed because variation was negligible.) Bilateral measurements were recorded from the right-hand side.

### Morphological analyses

Sexes were analyzed separately. The pooled within-group coefficients from the least-squares regressions of log_10_ body dimensions against log_10_ SVL were used to adjust each body dimension to its value at the overall mean log_10_ SVL. Discriminant function analyses (DFA) were computed on these size-adjusted values, with individuals grouped by sampling locality. This method provides functions that maximize among-group relative to within-group variation, and therefore allows investigation of whether body dimensions differ between regions. DFAs were also applied to the three scalation characters.

### DNA sequencing

DNA was extracted from tail-tips using a standard phenol–chloroform procedure. Primers were designed or used from previous studies (Appendix S2). The following mtDNA fragments were amplified: (i) 737—795 bp (depending on primers used) of NADH dehydrogenase subunits 1 and 2 and three intervening tRNAs (this fragment will be referred to as “NADH”) and (ii) 655 bp from the cytochrome b gene (*cytb*). Two nuclear gene fragments were also amplified: (i) 371 bp of the c-*mos* gene and (ii) 713 bp of the RAG-1 gene. Polymerase chain reaction (PCR) products were sequenced by GATC Biotech (Konstanz, Germany) and Source BioScience (Nottingham, U.K.). Wherever possible, several individuals were sequenced from each site for the NADH (total number of individuals sequenced, *n* = 62) and c*-mos* (*n* = 84) fragments in order to gain insights into within-site genetic diversity. One or two individuals were sequenced per site for *cytb* (*n* = 21) and RAG-1 (*n* = 20).

### Genetic relationships and clustering

Median-joining networks were used to portray relationships among sequences (software: Network 4.6.0.0, Fluxus engineering). Multilocus genetic structure within *C. mionecton* was investigated using the program BAPS (“Bayesian Analysis of Population Structure”; Corander et al. 2003). When using sequence data, BAPS has advantages over other structuring programs because it can treat individual base positions within a locus as linked loci, while treating different loci as independent. Forty-six specimens that had been sequenced for both mtDNA and c*-mos* were selected for this analysis. Twenty of these specimens had also been sequenced for RAG-1 and these sequences were also included in the analysis. No prior information on geographic location was specified. Replicated runs were performed on different values of *k* from 2 to 10.

### Migration and isolation

The Bayesian Markov Chain Monte Carlo (MCMC) approach implemented in the program IMa2 (Hey and Nielsen 2007; Hey 2010) was used to jointly examine migration and isolation between the primary genetic clusters detected by BAPS. Other coalescent analyses used to analyze these data (see below) assume no migration, and so it was important to evaluate this assumption. The mtDNA was represented by all NADH sequences. Nuclear loci were represented by all c-*mos* and RAG-1 sequences (note that, unlike many phylogenetic methods, different specimens can be sampled for different loci). We obtained the multilocus maximum likelihood values of the per locus Watterson's estimator of population mutation rate (see Ross-Ibarra et al. 2009). This is estimated as θ = N*μ* for mtDNA and θ = 4N*μ* for nuclear loci, where N is the population size and *μ* is the mutation rate per generation, for the genetic clusters and for each locus. Geometric means across loci were obtained for each cluster, and the largest of the two (θ_MAX_) was used to specify upper bounds on uniform prior distributions. Specifically, population sizes were specified from the uniform distribution, U(0,5θ_MAX_), splitting time, *t* (in generations) was specified from U(0,2θ_MAX_), and migration rates per mutation (m) were specified from U(0,2/θ_MAX_). The rationale for using these prior upper bounds is outlined in the IMa2 documentation (http://lifesci.rutgers.edu/∼heylab/ProgramsandData/Programs/IMa2/Using_IMa2_8_24_2011.pdf). The mutation rate for the NADH sequence (per locus per year) was calibrated using a 1.1 Ma divergence between *Chalcides* from the Canary islands of La Gomera and El Hierro (see next section; Brown and Pestano 1998; Carranza et al. 2008). This enabled estimation of parameters on demographic scales. The MCMC chain was run for 1.505 × 10^7^ generations, with a sample interval of 100, and the first 50,000 generations were discarded. Twenty simultaneous chains were run, with a geometric heating model used for chain swapping. Marginal posteriors were estimated from the first half of the run and compared with those from the second half to ensure the entire chain was sampling from the correct posterior and runs were repeated several times from different starting points and convergence on the same posterior assessed.

Probabilities of differences between population sizes and migration rates were obtained by pairwise comparisons of posteriors. Population migration rates were obtained as 2NM (where M = m*μ* and is the probability of migration in a specific direction, per gene copy per generation). A likelihood ratio test (LLR) (Nielsen and Wakeley 2001) was used to investigate whether 2NM differed significantly from zero.

### Species delimitation

The program BPP (Yang and Rannala 2010) was used to examine delimitation of genetic clusters identified using BAPS. We used the two primary genetic clusters to map specimens onto the two species guide tree used in the analysis. The rjMCMC algorithm in BPP provided a posterior probability for the two species tree. The loci used in the IMa2 analyses were used. Population size parameters (Θ) were specified from a gamma prior, G(1,10). The age of the root in the species tree (τ_0_) was assigned from the gamma prior G(1,10), while the other divergence time parameters were assigned from a Dirichlet prior (Yang and Rannala 2010, equation 2). It should be noted that descriptions of probability distributions match the definitions for individual programs in this report and are not necessarily consistent between programs. The priors were determined after preliminary analyses in which τ_0_ and Θ were specified using a range of gamma distributions, from G(1,10) to G(1,2000). Not surprisingly, this led to slightly different posterior estimates of these parameters, although the posterior probability for the two species model was unaffected. Analyses were run several times to check for consistency (2 × 10^5^ iterations of the chain, sampling interval of 15, burnin = 10,000).

### Divergence times

The Bayesian methodology implemented within *BEAST (ver. 1.6.1) was used for robust estimation of divergence times between genetic clusters identified by BAPS (Drummond and Rambaut 2007). The method combines tree inference, phylogenetic dating, and the coalescent and allows time constraints to be imposed on the species tree rather than on individual gene trees (through the addition of commands to BEAUTI-generated xml input files). NADH and *cytb* sequences from *C. mionecton* from all sites (*n* = 21), together with the nine Canary Island *Chalcides* described above, were included in the analysis. Nuclear loci showed negligible diversity across the Canary Island taxa and so we used only the mitochondrial locus for the phylogenetic analyses (in phylogenetic dating, slowly evolving or short sequences often have little influence on the posterior, Brown and Yang 2010). The sequences were divided into four sequence partitions corresponding to the three codon positions (across all mitochondrial genes) and the tRNA region of the NADH sequence.

Species were defined as follows: (i) Northern genetic cluster of *C. mionecton*, (ii) Southern *C. mionecton*, (iii) *C. sexlineatus* (Gran Canaria), (iv) *C. viridanus* (Tenerife), (v) *C. coeruleopunctatus* (La Gomera), (vi) *C. coeruleopunctatus* (El Hierro). Time priors were placed on the species tree, with 1 time unit = 10 Ma. The general prior on divergence times was specified using a Birth–Death model, with the birth–death rate specified from the uniform distribution U(0,+∞) and death rate relative to birth rate specified from U(0,1). The times of the most recent common ancestors (tMRCAs) of different species groups were constrained using geological information. The oldest emerged parts of the Canary island of El Hierro have been dated at 1.12 Ma with the island likely to have been colonized soon after its subaerial emergence (Brown and Pestano 1998). The gamma distribution G(110,0.001) was therefore used to quite tightly constrain the *C. coeruleopunctatus* (La Gomera, El Hierro) node. Two other nodes were also constrained albeit more loosely. The age of the root was specified from the gamma distribution G(6.8,0.1), and the ancestor of all Canary Islands sequences was specified from G(5.3,0.1), following previously published estimates (Brown and Pestano 1998; Carranza et al. 2008).

A relaxed lognormal clock was used for rates on the gene tree, with the mean assigned from a G(1,1) distribution, and the standard deviation from the exponential distribution Exp(0.33), for all partitions. The HKY+G model was specified with kappa parameters assigned from G(1,10) and alpha parameters from U(0,20), for all partitions. Monophyly constraints were imposed on sequences from the same island, on La Gomera and El Hierro together, on all Canary Island *Chalcides,* and on each of the two forms of *C. mionecton* (see Brown and Pestano 1998, Carranza et al. 2008).

MCMC chains were run for 3 × 10^7^ generations with a sampling interval of 1000, and the first 3000 samples were discarded as burn-in. Convergence diagnostics as well as posteriors on the gene and species trees were obtained using Tracer (v1.4.1) (Rambaut and Drummond 2007) and TreeAnnotator (v1.6.1)(Drummond and Rambaut 2007).

To date, it has been common to use Bayesian phylogenetic methods to date sequence divergence. In these analyses, time calibrations are applied to sequences and sequence divergence times are estimated. We also used this approach (we refer to it as the BEAST analysis) for comparison with the *BEAST phylogenetic–coalescent analysis described above. The priors within the analysis were the same as for the *BEAST analysis, but applied to tMRCAs of the sequences within the relevant species.

## Results

### Morphology

All individuals obtained from sites south of Cap Rhir (sites 11–16) were found to have five digits on the manus and the pes, except for one individual at site 16 which possessed four digits on the pes only (Fig. 1). Individuals captured from Cap Rhir (site 10) north to site 7 had four digits on the manus and the pes. Four- and five-digit individuals were detected in the most northern group of samples from sites 1–6: both forms were detected at sites 1 and 6, while only five-digit forms were detected at sites 2, 3, 4, and 5.

For female body dimensions, the first discriminant function (DF1) represented 35.4% of the total variation among sites, while the second function (DF2) represented 28.1%. Values were similar for males: DF1 = 36.2%, DF2 = 24.6%. Patterns of pooled within-group correlations between body dimensions and standardized DFs were quite similar between sexes (Table 1). However, female DF1 was most highly correlated with HL and ST, while male DF1 was quite highly correlated with several body dimensions (SVL, BWT, FLL, FTFL, ST). DF2 was most highly correlated with BWT for both males and females.

**Table 1:** Partial male and female structure matrices, showing correlations between the first two discriminant functions (DF1 and DF2) and each body dimension (which were transformed/adjusted as described in the text)

	Females		Males	
		
Body dimension	DF1	DF2	DF1	DF2
SVL	−0.180	−0.088	0.338	0.016
HL	0.526	−0.273	−0.162	−0.130
HD	0.122	−0.107	−0.213	−0.146
HW	0.252	−0.048	−0.041	−0.113
JL	0.063	−0.415	0.062	−0.123
ST	0.602	−0.261	−0.309	−0.336
SHL	0.211	−0.256	−0.281	0.333
BWT	0.290	0.607	−0.324	0.646
FLL	0.303	0.176	−0.334	0.322
RLL	−0.116	0.145	−0.089	0.497
FTFL	−0.325	0.267	0.322	0.476
FTHL	−0.314	0.272	0.084	0.146

SVL, snout–vent length; HL, head length; HD, head depth; HW, head width; JL, jaw length; ST, snout–thorax; SHL, snout–hind limb length; BWT, body width at thorax; FLL, fore–limb length; RLL, rear–limb length; FTFL, fourth toe on fore–limb length; FTHL, fourth toe on hind limb length.

**Figure 2:**
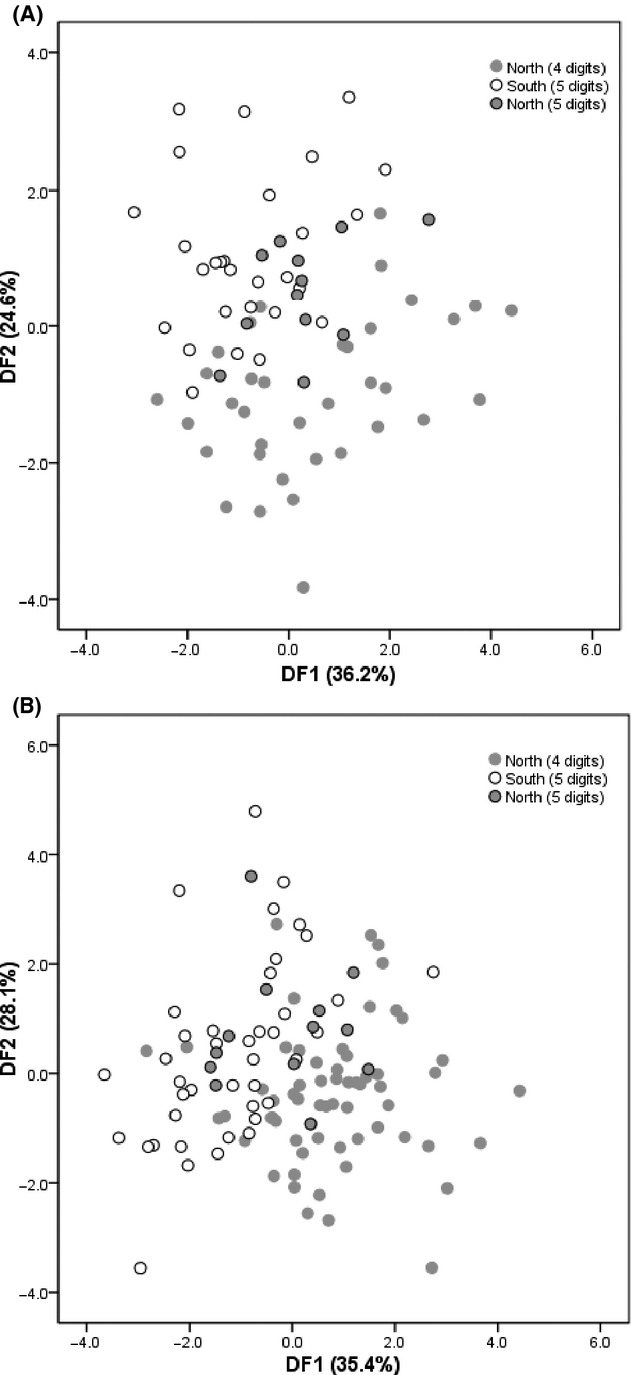
Discriminant function scores (DF1 and DF2) for body dimensions, and the proportions of variation that the functions represent, for males (A) and females (B). Markers indicate the northern form (sites 1–10, black circles) and the southern form (sites 11–16, gray circles). Northern individuals with five digits are also indicated.

The northern populations with one or more five-digit individuals were ostensibly intermediate between the remaining four-digit northern populations and the five-digit southern group (Fig. 2). There was considerable overlap between the two groups. Geographic concordance between digit number and DFs was investigated using correlation between population means (sites 2–5 grouped). Male DF2 (*r* = 0.76, *P* = 0.003) and female DF1 (*r* = −0.728, *P* = 0.005) were significantly associated with digit number, but correlations were not significant for male DF1 (*r* = 0.45, *P* = 0.126) or female DF2 (*r* = 0.39, *P* = 0.194). DFAs on males and female scalation characters revealed no differences between northern and southern forms (results are not shown for conciseness).

### Networks and genetic clustering

**Figure 3:**
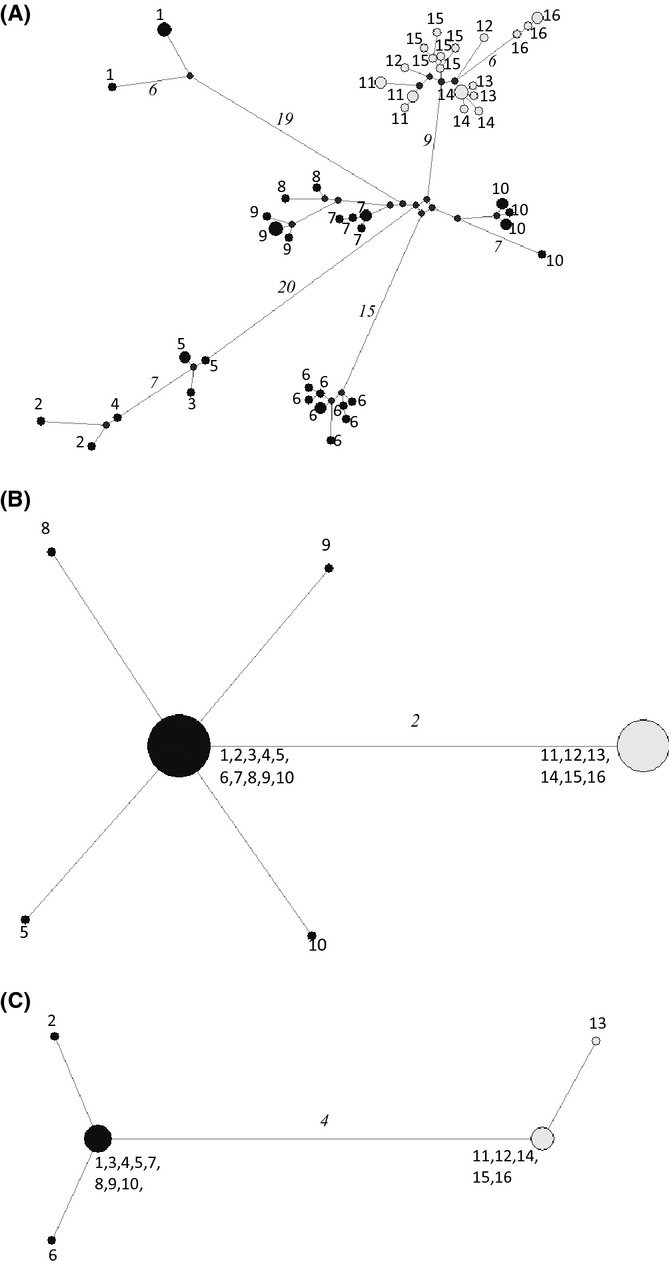
Median-joining networks showing relationships between haplotypes for NADH (A), c-*mos* (B), and RAG-1 (C). Haplotypes are represented as gray (southern) or black (northern) circles, with diameters proportional to frequencies and labeled by sites at which they were found. Lengths of the longer branches of each network (in mutational steps) are given in italics. Small dark circles with no labels are median vectors.

For mtDNA, substantial numbers of mutational steps separate *C. mionecton* from different regions (Fig. 3A). The *cytb* and NADH networks were similar and so only the latter is shown. The five-digit southern form is confined to one branch. However, large numbers of mutational steps also separate haplotypes from different northern sites. The two nuclear loci both show a clear north–south pattern of divergence that corresponds exactly to the northern and southern forms described here (Fig. 3B and C). Numbers of mutational steps separating the different forms are much lower than for mtDNA (c-*mos*: 2, RAG-1: 4).

**Figure 4:**
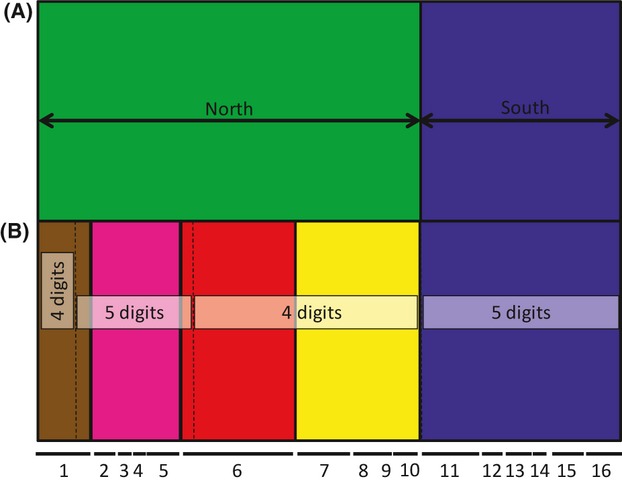
BAPS clustering of NADH, c-*mos,* and RAG-1 loci. It shows the assignment of individuals to clusters when *k* = 2 (A), and the assignment when *k* = 5 (the value of *k* with the highest likelihood) (B). The numbers at the bottom of the figure are the site numbers ordered from north (1) to south (16), as shown in Figure 1. Genetic clusters correspond to geography rather than morphotypes.

Assignment of individuals to *k* = 5 genetic clusters by BAPS provided the highest log likelihood (lnL = −1507.5). Higher values of *k* produced decreasing log likelihoods (*k* = 6, lnL = −1552.2; *k* = 7, lnL = −1615.6; *k* = 8, lnL = −1675.2; *k* = 9, lnL = −1737.02). However, all analyses assigned all southern individuals to a single cluster, that is, an increase/decrease in the value of *k* simply increased/decreased the number of northern clusters (individuals from sites 1–10) (Fig. 4). This is attributable to the high mtDNA diversity within the north, given the relative lack of within-region nuclear diversity. Because the optimal number of clusters appeared dependent on this single marker, it was sensible to focus on the primary north–south genetic clusters. To confirm this, we also ran analyses in which *k* = 2. This produced a lower likelihood (lnL = −1914.1) but the two genetic clusters represented northern individuals from sites 1–10, and southern individuals from sites 11–16 (Fig. 4).

### Migration and isolation

**Figure 5:**
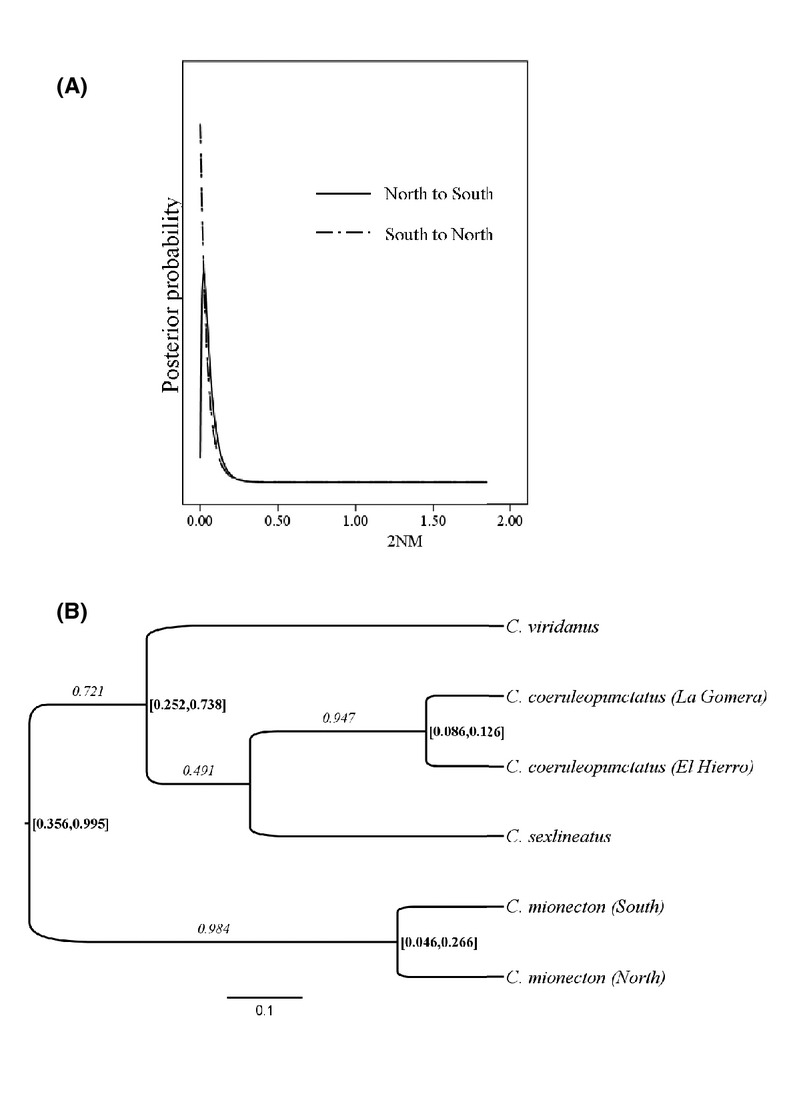
Coalescent analyses. Posterior distributions of population migration rates (2NM) between northern and southern forms are shown (forwards in time) (A). The consensus species phylogeny (B) shows 95% posterior intervals for species divergence times (where node support >0.5; 1 time unit = 10 Ma), and posterior node support on the branch above each node.

To facilitate interpretation, we will report on migration rates in the natural (forward) direction of time rather than coalescent time. Population migration rates from the south to the north are negligible (Fig. 5A), with a posterior mean of 0.048 and a 95% highest posterior density interval (HPD) which included zero (0.000, 0.150). The hypothesis of no migration was supported by the LLR test (*P* > 0.05). Population migration rates from the north to the south were also very low (mean = 0.060, 95% HPD [0.000, 0.158])(Fig. 5A), but here, the hypothesis of no migration was rejected by the LLR test (*P* < 0.001). Marginal posteriors on current population size parameters (θ) were represented by single peaks. For the southern form, θ_S_ was small: posterior mean = 0.401, 95% HPD (0.125–0.731), compared with θ_N_ (northern form): posterior mean = 1.007, 95% HPD (0.390–1.730). The difference between θ_S_ and θ_N_ was significant (Pr [θ _N_> θ _S_] = 0.997). Various exploratory analyses with different priors indicated that the data were relatively uninformative about the ancestral population size parameter, θ_A_, or the population splitting time.

### Species delimitation

The posterior probability of the north–south species delimitation was 1.00. As for IMa2, the posterior on Θ for the northern form indicated a larger population size (mean 0.0268, 95% HPD [0.0192, 0.0365]), compared with the southern form (mean 0.0096, 95% HPD [0.0059,0.0147]). Note that BPP and IMa2 are parameterized differently and so parameters are not directly comparable between programs. The posterior mean and 95% HPD for τ_0_ were 0.0064 (0.0038, 0.0097).

### Divergence times

The *BEAST coalescent analysis provided strong posterior support for the *C. mionecton* (North, South) species node (*P* = 0.98). The corresponding posterior mean speciation time was 1.43 Ma (95% posterior interval: 0.46–2.66 Ma)(Fig. 5B). Comparison of sequence divergence times with species divergence times is complicated by topological uncertainty in the mtDNA gene tree and discordance with the species tree. The posterior mean on the tMRCA of all *C. mionecton* sequences was 2.36 Ma, with a 95% posterior interval of (1.42, 3.60), which was approximately 1 Ma older than the posterior north–south speciation time within *C. mionecton* (see above). However, this does not represent a north–south split between sequences. The ancestral node that is common to the two mtDNA lineages, comprising sequences from northern (7–10) and southern sites (11–16), is quite well supported (*P* = 0.94). It has a posterior mean and posterior interval of 1.58 and (0.94, 2.46) Ma, respectively, which is quite similar to that obtained under the phylogenetic–coalescent analysis.

## Discussion

Species delimitation can require a combination of genetic, morphological, and biogeographical information in order to be convincing. Here, coalescent analyses of genetic data alone demonstrate negligible gene flow between northern and southern groups of contiguously distributed populations, indicating that they form separate metapopulation lineages (de Quieroz 2007). Integration over gene trees from three loci provides strong statistical support for the species delimitation. We also show that this splitting event is likely to have occurred during the Pleistocene, or possibly late Pliocene. Comparisons of recognized sister species of reptiles have suggested that speciation events that connect extant sister taxa are generally older than this, often coinciding with the early Pliocene or late Miocene (Avise 2000). However, the taxa examined here were originally recognized as subspecies, not species. A consequence of widespread application of the approach used here could be species inflation because statistical evidence allows delimitation of more closely related forms. This will cause a concomitant decrease in the average timing of speciation between sister species.

It is notable that the mtDNA tree shows evidence of incomplete lineage sorting between northern and southern species and fails to provide unequivocal support for the delimitation. In contrast, both nuclear loci show clear north–south sorting, despite a much slower substitution rate. Although microsatellite nuclear markers have been widely used in intraspecific studies because of their elevated evolutionary rates, here we show that more conserved nuclear sequences can also be useful when analyzed within a coalescent framework. Another important finding was that clear delimitation was achieved using just three loci. This reinforces the finding that coalescent analyses with just a few loci can have considerable statistical power to detect speciation when multiple individuals are available per population (Yang and Rannala 2010; Zhang et al. 2011).

Until recently, many phylogeography studies have used phylogenetic dating of sequence divergence times (Leaché and Mulcahay 2007; Brown and Yang 2010). We show that estimation of speciation times instead of sequence divergence times appears to considerably influence estimation of the timing of the north–south split. The speciation time (posterior mean 1.5 Ma) under the phylogenetic–coalescent analysis postdates the oldest sequence divergence time in the *C. mionecton* lineage by almost 1 Ma. It postdates the north–south mtDNA sequence divergence time from another phylogenetic study (Carranza et al. 2008) by an even greater margin. This is not entirely expected. Under the phylogenetic approach, constraints are placed on sequence divergence times and so predate speciation times. This incorrect calibration might be partially compensated by the fact that the posteriors on (uncalibrated) nodes of interest also represent sequence divergence and not speciation times. However, comparison between the two approaches was complicated by incomplete lineage sorting of mtDNA. In fact, the sequence divergence between the reciprocally monophyletic northern and southern sequences (the second most basal node within the *C. mionecton* mtDNA tree) is dated at 1.6 Ma which is not too different from the phylogenetic–coalescent date for divergence of northern and southern forms. While this highlights important differences between the dating approaches, detailed future comparisons between methods would be more informative in cases where it is appropriate to use a single topology for each analysis, rather than integrating over the range of possible topologies.

In *C. mionecton,* we find that digit number varies in a quite complex manner with five digits being more common throughout the far north of the range than previously thought (Schleich et al. 1996; Bons and Geniez 1996) as well as being polymorphic in some populations. This pattern is also quite concordant with variation in body dimensions. Within-population variation in morphological characters is substantial relative to between-population variation even for the discrete digit number character. This does not readily allow delimitation of populations into two taxa. Furthermore, the pattern of morphological differentiation is discordant with the two species delimitation.

It is interesting to speculate why some individuals from the most northerly populations should show morphological similarity with the most southern populations, despite delimitation of northern and southern individuals into discrete genetic clusters. The most parsimonious explanation is that the ancestral state in this species is five digits. Other morphological states found in the extreme north and south, such as a wider thorax, would also be considered ancestral but for clarity we focus our argument on digit number. The sister group to *C. mionecton* has five digits (Carranza et al. 2008) which would support this hypothesis. (Note that Brandley et al.'s (2008) interesting analysis of digit reduction in skinks infers an ambiguous ancestral state for *C. mionecton*, but they erroneously defined it as a three-digit species.) One hypothesis is that the four-digit morphology arose due to mutations that appeared in the northern form after N–S speciation. Gene flow between northern populations allowed these mutations to spread north, while restricted gene flow between species impeded southern movement. This scenario suggests that these morphological differences originated recently. However, it does not explain why the distribution of the four-digit morphology is interrupted by a northern area in which only five-digit individuals were found. It has been proposed that digit loss is evolutionary labile, with complete loss of digits over approximately 3.5 Ma in some Australian skink lineages (Skinner et al. 2008). Rapid evolution might indicate that digit number is determined by quite a simple genetic system. The Australian lizard genus *Hemiergis* shows interspecific patterns of digit number reduction that are similar to those for *C. mionecton* in which the phalanges that comprise the fifth digit are lost but metacarpals/metatarsals remain (Shapiro et al. 2003). This seems to be determined by regulatory genes that influence the duration and location of expression of the *Sonic hedgehog* (Shh) gene.

This paper does not aim to reconstruct the historical biogeography of *C. mionecton*. However, it does provide information on current distributions and divergence times. There was no evidence of unsuitable habitat between the southernmost four digit sample from Cap Rhir and the adjacent five-digit sample from Taghazoute (separated by a distance of around 25 km), so it is probable that the two forms come into contact without significant hybridization. Bons and Geniez (1996) reported syntopy of four- and five-digit forms at Cap Rhir, but we found no evidence to corroborate this. Our analyses suggest that a historical event led to divergence between forms which interrupted gene flow between them. This appears more likely than the hypothesis that divergence is due to an ongoing restriction to gene flow mediated by a physical barrier such as the Atlas Mountains, as suggested by Schleich et al. (1996). Other North African lizards show evidence of phylogeographical divisions associated with the Atlas range (e.g., Brown et al. 2002; Harris et al. 2007; Perera and Harris 2010). Levels of sequence divergence differ greatly between species, and so different evolutionary scenarios for different species appear likely. What might have caused population isolation in the Pleistocene is unknown. This was an important period for speciation of many African groups due to climatic fluctuations that affected vegetation patterns (e.g., deMenocal 1995; Swart et al. 2009; Trauth et al. 2009; Voje et al. 2009). The hypothesis that there was a break in suitable habitat, which led to fragmentation of *C. mionecton* populations, would appear plausible.

To summarize, we are able to examine key features of the speciation process, namely timing of divergence and gene flow between lineages, using only a small number of loci, two of which have low substitution rates. Bayesian methods provide strong statistical support for divergence of two lineages in the Pleistocene after which very few genes were exchanged. Previously described subspecies of *C. mionecton* therefore appear to represent good species under the unified species concept*,* but these species are not diagnosed by digit number or other external morphological characteristics.

## Supporting Information

**Appendix S1.** Site names and numbers of individuals sequenced/measured.

**Appendix S2.** Primers. All primers were all designed for the current study, except where indicated.

**Appendix S3.** Data for all three loci (mtDNA, c-*mos*, RAG-1) are provided in the file “Threeloci.txt.” Labels provide a site and a specimen identifier, for example, “8.02∧1” is specimen 2 from site 8 (see Fig. 1). The “∧1” suffix can be ignored but is useful if you run BPP on these data. The mtDNA data used for the estimation of divergence times are provided in MEGA format in the file “MtDNA.meg.” Data partitions (ND1, ND2, tRNA, cytochrome b) are shown together with codon positions within these partitions, where applicable. *C. mionecton* sequences are labeled by site and individual identifiers (as above), whereas *Chalcides* from other Canary Islands are labeled by the names of their collection sites (see Materials and Methods).
