# Supplementary material for: Species delimitation and digit number in a North African skink
Source: Ecol Evol. 2012 Oct 24;2(12):2962–73. doi: 10.1002/ece3.326 (PMC3538992; doi:10.1002/ece3.326)
Supplement: Supplementary file 1 [file ece30002-2962-SD1.docx]

Supplement S1. Site names and numbers of individuals sequenced/measured.

| **Site** | **ND1&2** | **Cytb** | ***c-mos*** | **RAG-1** | **Morphology** |
| --- | --- | --- | --- | --- | --- |
| 1. Port Lixus | 4 | 2 | 9 | 2 | 20 |
| 1. Oulaid Abdellah Al Dhirya | 2 | 2 | 2 | 1 | 2 |
| 1. Kenitra | 1 | 1 | 1 | 1 | 3 |
| 1. Chelihate | 1 | 1 | 1 | 1 | 0 |
| 1. Mehdia Plage | 3 | 1 | 8 | 1 | 13 |
| 1. Skhirat Plage | 9 | 2 | 9 | 1 | 11 |
| 1. Lamchat (nr. Bit el Mekki) | 5 | 1 | 7 | 1 | 16 |
| 1. Cap Beddouzza | 2 | 1 | 5 | 1 | 19 |
| 1. Oum el Aïon | 5 | 1 | 3 | 1 | 21 |
| 1. Cap Rhir | 6 | 1 | 6 | 2 | 19 |
| 1. Taghazout | 5 | 2 | 6 | 2 | 5 |
| 1. Nr. Taroudant | 2 | 2 | 2 | 1 | 3 |
| 1. Admin (nr. Agadir airport) | 4 | 1 | 5 | 1 | 20 |
| 1. Takat | 3 | 1 | 7 | 1 | 13 |
| 1. Tassila | 6 | 1 | 5 | 1 | 15 |
| 1. Sidi Ifni | 4 | 1 | 7 | 2 | 12 |
