# Supplementary material for: Species delimitation and digit number in a North African skink
Source: Ecol Evol. 2012 Oct 24;2(12):2962–73. doi: 10.1002/ece3.326 (PMC3538992; doi:10.1002/ece3.326)
Supplement: Supplementary file 2 [file ece30002-2962-SD2.doc]

Appendix 2. Primers. All primers were all designed for the current study, except where indicated.

| Sequence | Forward primer | Reverse primer |
| --- | --- | --- |
| ND1&2 | 5’- CTAGCTGAAACCAACCGAGCCCC-3’ | 5’-GAAGTATTTTGTTGCGGCCTC-3’ |
| ND1&2 (alternative) | 5’-TTCTGCTCTTGACCTCTAGCAA-3’ | 5’-GCAGCTTGGGTGAGGAAGTA-3’ |
| cytb | 5’- GACCCACAACCTACGAAAAAC-3’ | 5’-CCTAGCAGGTCTTTGTA-3’ |
| c-*mos*^1^ | 5’-GCGGTAAAGCAGGTGAAGAAA-3’ | 5’-TGAGCATCCAAAGTCTCCAATC-3’ |
| RAG-1^2^ | 5’-TCAGATGGACACAGCACTGA-3’ | 5’- GTCTTGGGAAGCTACTGCCC-3’  5’-TACTGCCCCTCTTGTCGCTA-3’ |

^1^Saint et al., 1998. Mol.Phy. Evol. 10: 259-263.

^2^Both reverse primers used in PCR.
